# Metabolic Syndrome as a Risk Factor Among Lebanese Patients with Substance Use Disorder Undergoing Treatment for Recovery Through Rehabilitation or Opioid Substitution Treatment

**DOI:** 10.3390/clinpract14060210

**Published:** 2024-12-10

**Authors:** Nadine Mahboub, Elissa Ayoub, Carine Mounzer, Tatiana Kate Baltagi, Dimitrios Papandreou, Nanne de Vries, Rana Rizk

**Affiliations:** 1 Department of Nutrition and Food Sciences, Faculty of Arts and Sciences, Lebanese International University, Beirut P.O. Box 146404, Lebanon; 2 Department of Nutrition and Dietetics, Faculty of Public Health Branch 2, Lebanese University, Beirut P.O. Box 6573, Lebanon; elissa.ayoub.1@st.ul.edu.lb (E.A.); carine.monzer@st.ul.edu.lb (C.M.); 3 Department of Biochemistry, College of Arts and Sciences, Case Western Reserve University, Cleveland, OH 44106, USA; tkb43@case.edu; 4 Department of Clinical Nutrition & Dietetics, College of Health Sciences, University of Sharjah, Sharjah P.O. Box 27272, United Arab Emirates; dpapandreou@sharjah.ac.ae; 5 Department of Health Promotion, CAPHRI School for Public Health and Primary Care, Maastricht University, P.O. Box 616, 6200 LK Maastricht, The Netherlands; nanne.devries@maastrichtuniversity.nl; 6 Department of Nutrition and Food Science, Faculty of Arts and Sciences, Lebanese American University, Byblos P.O. Box 36, Lebanon; rana.rizk01@lau.edu.lb; 7 Institut National de Santé Publique, d’Epidémiologie Clinique Et de Toxicologie (INSPECT-LB), Beirut P.O. Box 14404, Lebanon

**Keywords:** substance use disorder, metabolic syndrome, rehabilitation, opioid substitution treatment, Lebanon

## Abstract

**Background/Objectives:** Data about metabolic syndrome (MS) in people who use drugs (PWUD) undergoing treatment for recovery are limited. We aimed to explore the extent of the MS and its predominant components and determinants in a sample of PWUD undergoing treatment for recovery through rehabilitation or opioid substitution treatment (OST) in Lebanon. Furthermore, we investigated the effect of each treatment modality on the MS; **Methods:** This was a cross-sectional study, in which demographics and treatment-related, nutritional, and biochemical data of the participants were collected. MS was defined according to the American Heart Association and the National Heart, Lung, and Blood Institute (AHA/NHLBI) criteria. Descriptive statistics were presented, and bivariate and multivariate analyses were conducted; **Results:** A total of 155 male subjects with the following characteristics were included: OST: n = 80; rehabilitation: n = 75; mean age: 32.53 ± 8.39 years; mean body mass index (BMI): 27.41 ± 4.99 Kg/m^2^; mean duration of treatment: 18 months. More than half of the sample had low HDL-C (56.8%) and/or elevated blood pressure (51.6%), 42.9% had elevated WC, 21.9% had elevated TG, and 12.3% had elevated FBS. Furthermore, 7.2% of the sample had no components of the MS, 29.2% had one component, 40.9% had two components, 16.9% had three components, and 5.8% had four components. MS was identified in 22.7% of the sample. Higher age was associated with higher odds of being diagnosed with MS (OR = 1.072; 95% CI: 1.021–1.126), whereas higher duration of current treatment was associated with lower odds (OR = 0.969; 95% CI: 0.944–0.995); **Conclusions:** MS and its components are prevalent in PWUD undergoing treatment for recovery. Routine screening and preventive measures are essential to prevent metabolic syndrome, particularly among older people and treatment newcomers.

## 1. Introduction

Substance use disorder (SUD) consists of an uncontrollable urge to seek legal or illegal drugs, alcohol, or unprescribed medications. SUD causes immense adverse socio-economic and health effects on individuals and society [1,2]. In the past decades, medication-related problems, such as medication errors, misuse, and abuse, mainly involving opioids as analgesics, have reached significant proportions leading to increased opioid dependence, adverse drug reactions (ADRs), and related fatalities [3]. Several studies indicate that a significant portion of ADRs and medication errors are preventable, highlighting the importance of implementing preventive measures and safety protocols in health care facilities and palliative care settings [4]. To prevent drug toxicity, as well as withdrawal and dependence problems, the rational and safe use of medications includes weighing all potential advantages and disadvantages, applying them only to conditions that are indicated, and using them for the shortest amount of time and at the lowest dosage [3,5]. Globally, over 35 million people suffer from SUD, requiring treatment for recovery [6]. Two main types of treatment are offered: opioid substitution treatment (OST) in outpatient centers using opioid agonists, such as buprenorphine and methadone [7], and rehabilitation aiming for complete abstinence in supervised inpatient treatment centers.

Despite the enormously high rates of mortality among PWUD, a great part of this population would reach an age at which the main causes of illness or death are chronic diseases [8]. Rather than tending to concerns related to drugs and infections, the health needs of people with SUD or advanced treatment stages are more likely related to noncommunicable diseases [8,9]. Accordingly, there is an additional burden of morbidity among PWUD undergoing treatment for recovery, as causes of death shift from infectious to chronic diseases occurring later in life [10].

MS is a complex disorder defined by a group of abnormalities associated with insulin resistance, hypertension, obesity, hyperinsulinemia, hypertriglyceridemia, and low HDL-C [11]. These interconnected factors increase the risk of cardiovascular, atherosclerotic diseases, and type 2 diabetes [11,12]. Studies investigating MS in PWUD and those undergoing treatment for recovery are extremely scarce [13,14]. A history of drug use was identified as a risk factor for MS, especially when combined with energy deficiency and poor nutritional status [15]. This may be explained by various mechanisms, including increased cell damage, excitotoxicity, decreased cellular antioxidant power, and reduced energy production [15,16]. The weight regained during treatment associated with increased abdominal adiposity, WC, and insulin resistance could also foster the development of MS [17].

SUD treatment presents an opportunity to holistically address health among this population group. However, SUD patients have shown persistent unhealthy lifestyle behaviors, even while being treated [18,19]. The low life expectancy and negative health outcomes among PWUD undergoing recovery treatment were shown to be related to cardiometabolic abnormalities [20] arising from modifiable risk factors like sedentary lifestyle, poor sleep quality, cigarette smoking, and poor dietary habits [9,21,22]. Moreover, the treatment of SUD has been linked to several metabolic and endocrine abnormalities [23]; specifically, OST is associated with glucose metabolism abnormalities, particularly insulin resistance [24,25,26] and diabetes [27,28,29].

In Lebanon, SUD is a growing public health concern, in which substance use is highly prevalent and is associated with a high burden of disease [30,31]. The nation’s capacity to address mental health, SUD, and associated communicable and noncommunicable diseases has been strained as a result of the socio-economic disadvantage the country is facing, in addition to the significant number of refugees in recent years [32].

MS remains an overlooked risk factor among this group of populations. There is a paucity of published literature that has critically reviewed the epidemiology of MS among substance users and its association with various sociodemographic and clinical variables enhancing the understanding of MS among substance users. Studying MS among PWUD undergoing treatment for recovery in Lebanon presents a novel valuable area of research as there is a notable gap in studies examining the prevalence of MS among this population group. Moreover, Lebanon has a growing concern with substance use so this study can lead the path for the development of targeted interventions in treatment. Additionally, this research can provide a comparison point to other Middle Eastern countries or regions with similar cultural contexts offering a broader understanding of the problem. Hence, the aim of this study is to explore the extent of MS and its predominant components and their determinants in a sample of PWUD undergoing treatment for recovery in Lebanon. Moreover, it further investigates the effect of different treatment modalities (rehabilitation vs. OST) on MS as they vary in their pharmacological, behavioral, and lifestyle approaches [33].

## 2. Materials and Methods

### 2.1. Study Design and Population

This study is a cross-sectional study exploring the nutritional status and lifestyle practices of male PWUD undergoing treatment for recovery in OST and rehabilitation residential centers in Lebanon [34]. All treatment centers were approached; three out of four OST centers and four out of seven rehabilitation centers granted us entry permission. The inclusion criteria included (1) being Lebanese, (2) being aged 18 years and above, and (3) receiving treatment for more than one month. Female participants were not included in this study due to several reasons. Mainly, a limited number of rehabilitation centers in Lebanon accommodate females. Additionally, there is a fear of stigma among females receiving OST hindering their participation. This stigma can impact the availability of data and limit the ability to recruit diverse and adequate samples. Moreover, additional barriers, like childcare responsibilities or fear of legal consequences, hindered female participation in this study. Hence, the sample size of female participants was small, and this prevented us from depicting the effect of substance abuse on metabolic syndrome in this population. The exclusion of female participants can affect the generalized recommendations, as there is a risk of sex base bias caused by assuming that findings for men apply equally to women. This can lead to overgeneralized conclusions and the creation of guidelines that do not account for gender-specific differences. Future studies addressing these challenges taking into consideration culturally sensitive approaches that respect women’s privacy, ensure confidentiality, and provide safe and supportive environments for them to participate are warranted. The present study was conducted according to the guidelines of the Declaration of Helsinki, and the ethical approval of all procedures involving the study participants was obtained from the Lebanese International University’s Committee on Research Ethics (CRE) (case number: LIUIRB-180122-NB, on 3 July 2018). Written informed consent was obtained from all subjects prior to data collection.

### 2.2. Data Collection

Study parameters were collected by trained licensed dietitians in treatment facilities using the tools below in Arabic. Data collection required 40–50 min per participant.

- A questionnaire focusing on sociodemographic characteristics, medications, frequency of drug use, types of drugs used, duration of drug use, and the type of drug treatment chosen was used to assess demographics, medical history, and history of drug use and treatment.
- A 24 h recall questionnaire was used for the dietary assessment using the United States Department of Agriculture’s Multiple Pass Food Recall (MPR), which attenuates the recall bias. To provide complete information on food consumption and help determine portion sizes, the dietitian queried the participants several times during the interview. The participants’ daily energy and macronutrient intake were calculated from 24 h recalls using the Nutritionist Pro software food composition database (Nutritionist Pro, Axxya Systems, San Bruno, CA, USA, version 5.1.0, 2018). The database software was enhanced by the addition of an analysis of locally consumed foods and recipes. The values, after analyzing the data, were compared with US-based Dietary Reference Intake Tables, as recommended by the Institute of Medicine, because there are no sex- or age-specific Dietary Reference Intakes (DRIs) for Middle Eastern populations.
- Food addiction was assessed using the Yale Food Addiction Scale (YFAS), which includes 27 items assessing signs of addictive-like eating behavior, including tolerance and withdrawal, vulnerability in social activities, etc. [35]. This scale has a high internal consistency (Cronbach’s α in this study is 0.84). If three or more of the specified eating behaviors are shown during the past 12 months, food addiction is diagnosed.
- Physical activity level was assessed using the International Physical Activity Questionnaire (IPAQ), which assessed physical activity in the last seven days [36,37]. The questions encompassed four sections aimed to assess the regularity and time spent by the participants on vigorous physical activities, moderate physical activities, walking activities, and sitting. The metabolic equivalents of tasks (METs) are obtained by multiplying the total minutes spent in the corresponding activities by the frequency (days) and the constants of 3.3, 4, and 8 for light, moderate, and vigorous activity, respectively. The total MET value is calculated by summing up the MET values for all activities that were performed in spurts but were longer than 10 min. METs are categorized to have low, moderate, and high PA levels.
- Sleep quality was assessed using the Pittsburg Sleep Quality Index (PSQI) developed by Buysse et al. [38]. The PSQI is a validated questionnaire used to measure the quality and patterns of sleep in adults during the past month [38,39]. The first four questions assessed sleep initiation: the sleep latency or time needed to fall asleep, the time needed to wake up, the duration of actual sleep, and the awake time in bed. The remaining questions addressed the reasons for troubled sleep, the use of sleeping medication, daytime dysfunction, and the subjective sleep quality. To obtain the final score, answers were converted using an algorithm adopted from the developers of the questionnaire. Higher scores (≥5) indicate poor sleep quality, and lower scores (0–4.9) indicate good sleep quality.
- Nutrition knowledge was assessed using Consumer-Oriented Nutrition Knowledge (CoNKQ), which was adapted from Spillmann and Keller, consists of twenty items with a dichotomous (yes/no) answer type derived from consumer interviews and expert recommendations about healthy eating, and demonstrates good internal reliability (Cronbach’s α is 0.73). CoNKQ evaluates the knowledge of concepts and processes related to the following: nutrition and health, diet and health, diet and disease, major sources of nutrients, dietary recommendations, calories in food, health benefits of certain foods, and comparisons between different types of food. A score of less than 60% indicated poor knowledge.
- Biochemical parameters were analyzed in a laboratory certified by the Ministry of Public Health (MOPH) in Lebanon. A certified phlebotomist drew a blood sample of 5 mL after an overnight fast. The samples were centrifuged directly using a portable machine and transported to the laboratory using a thermally insulated box. Serum was analyzed for fasting blood sugar (FBS, mg/dL), cholesterol (mg/dL), high-density lipoprotein cholesterol (HDL, mg/dL), triglycerides (TG, mg/dL), aspartate aminotransferase (AST, IU/L), and alanine aminotransferase (ALT, IU/L) using a spectrophotometer and the Beckman Coulter (Unicel DXC 600) machine.
- Anthropometric measurements were taken by a licensed dietitian as follows: (1) height (cm), rounded off to 0.1 cm, was measured using a portable digital wall height scale without shoes; (2) weight (kg) was taken using a calibrated mechanical floor scale without shoes and light clothing; (3) body mass index (BMI) was calculated as the ratio of weight (kg) and height squared (m); and (4) waist circumference, rounded off to 0.1 cm, was taken using a girth measuring tape. Blood pressure (mmHg) was measured using a standardized mercury sphygmomanometer (ALPK2, Japan) while being seated after 5 min of rest, without prior smoking and exercise on that day. The mean of two consecutive measurements for each systolic blood pressure (SBP) and diastolic blood pressure (DBP), which were taken on the same arm within a 2 min interval, was used for analysis.

The Arabic version of the PSQI [40] was used, while the CoNKQ, YFAS, and IPAQ were translated back and forth by two independent bilingual expert translators. Moreover, the translated versions of these questionnaires were validated by administering them to a group of participants from other treatment centers. These results were not included in this report.

### 2.3. Metabolic Syndrome

MS was diagnosed based on the criteria set by the American Heart Association (AHA) [41] and the National Heart, Lung, and Blood Institute (AHA/NHLBI), which consist of any three of the following five criteria:

- Waist circumference ≥ 94 cm: the cut-off used for the Arab population.
- TG ≥ 150 mg/dL or being on drug treatment for elevated TG.
- HDL-C < 40 mg/dL in men or being on drug treatment for reduced HDL-C.
- Blood pressure ≥ 130 mm Hg SBP or ≥85 mm Hg DBP or being on antihypertensive drug treatment.
- FBS ≥ 100 mg/dL or being on drug treatment for elevated glucose.

### 2.4. Statistical Analysis

The Statistical Package for the Social Sciences (SPSS) version 21.0 was used for data entry, management, and analyses. Continuous data were reported as means and standard deviation (SD), and categorical data were reported as frequencies and percentages. The normality of the data was tested using the Shapiro–Wilk Test. An independent samples *t*-test was used to compare data for continuous variables with two groups with a normal distribution, and a Mann–Whitney U Test was used for variables with a skewed distribution. The chi-squared test was used to compare data for categorical variables.

Bonferroni correction was applied for multiple tests; the corrected *p*-value was obtained by dividing 0.05 by the number of variables to be tested (=23), yielding a *p* = 0.002 in the bivariate analysis. All the values that have a *p* ≤ 0.002 were considered statistically significant.

A logistic regression analysis was applied to assess the determinants of the MS and its components while adjusting for potentially confounding variables. No multicollinearity was present between the independent variables; thus, they are not highly correlated with each other. First, an exploratory bivariate analysis was conducted based on factors identified in the literature, such as age, duration of treatment, type of treatment, caloric intake, and other lifestyle factors associated with MS [15,42,43,44]. Variables with a *p*-value < 0.25 in the bivariate analysis were included in the regression model [45,46,47,48,49]. The chosen *p*-value < 0.25 will allow for the reduction of the initial number of variables in the model and the inclusion of the most important one while reducing the risk of missing main variables. Since the number of these variables exceeded 4 (10% of the group having the MS, n = 35), the respective effect sizes (ES) were calculated. The ES corresponded to Cohen’s d and Cramer’s V for continuous and categorical variables, respectively. The four variables with the highest ES were age (years), pre-treatment BMI (Kg/m^2^), duration of drug injection (years), and caloric intake per Kg of body weight (Kcal/Kg). However, since the duration of the drug injection was analyzed only among those who inject drugs, not the total sample, this variable was withdrawn. In consequence, the variables that were included in the model were age (years), duration of current treatment (months), pre-treatment BMI (Kg/m^2^), and caloric intake per Kg of body weight (Kcal/Kg). Moreover, five logistic regression analyses using the Enter method assessed the determinants of the components of the MS (yes vs. no). Odds ratios and 95% confidence intervals were calculated. For the bivariate and multivariate analyses, a *p*-value < 0.05 indicated statistical significance.

## 3. Results

### 3.1. Demographics, Medical History, and Drug Use History

In total, 369 people were approached, 214 agreed to participate in the study (response rate: 57.9%), and 187 (50.7%) met the inclusion criteria. Out of the 187 participants, 32 were excluded for the following reasons that hampered the diagnosis of MS: impossible blood collection as indicated by the phlebotomist (n = 17), no fasting (n = 7), and no consent for blood collection (n = 8).

Finally, 155 PWUD (42.0%) undergoing treatment for recovery (OST: n = 80; rehabilitation: n = 75) were included in the study. Basic demographic information, medical history, and drug use history of the sample are presented in Table 1. The mean age of the participants was 32.53 ± 8.39 years, 5.8% were illiterate, and the majority received at least an intermediate level of education. Half of the participants were unemployed (46.5%), with 72.3% being single at the time of the assessment. More than one-third of the participants used antipsychotic medications (37.4%), around one-quarter (22.6%) used antidepressants, and 21.3% used epilepsy/bipolar medications. The average age for drug use initiation among the participants was 16.57 ± 3.95 years. During their active drug use, half of the participants (48.4%) used and injected drugs concurrently, and 78.1% of them used drugs more than three times per day. Almost half of the participants (45.2%) were not previously treated for recovery. The average treatment duration of the participants was around 18 months and was significantly higher in the OST group.

### 3.2. Metabolic Syndrome and Its Components

**Figure 1:**
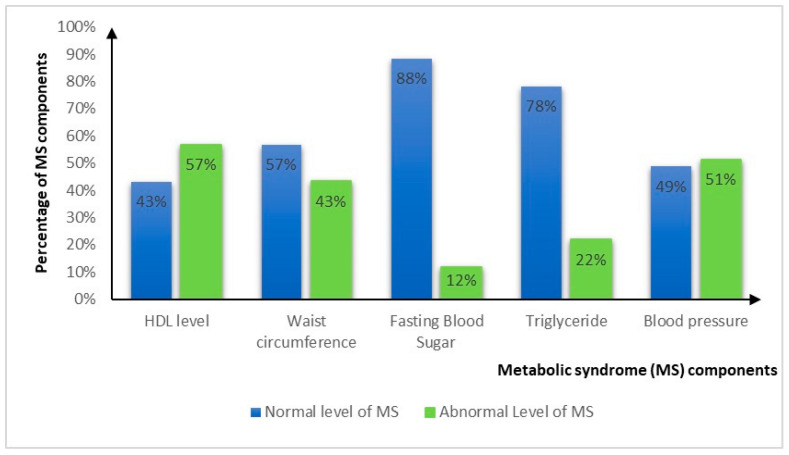
Proportions of the metabolic syndrome components in the total sample (n = 154). HDL-C: high-density lipoprotein cholesterol.

The biochemical parameters and anthropometric measurements of the participants are presented in Tables S1 and S2. Looking at the proportions of the MS, more than half of the sample had low HDL-C (56.8%) and/or elevated blood pressure (51.6%), 42.9% had elevated WC, 21.9% had elevated TG, and 12.3% had elevated FBS. Furthermore, 7.2% of the sample had no components of the MS, 29.2% had one component, 40.9% had two components, 16.9% had three components, and 5.8% had four components. Around one-quarter (22.7%, n = 35) of the sample were diagnosed with MS: 16.5% and 29.3% for OST and rehabilitation, respectively, with no statistical difference between treatment types (*p* = 0.057) Figure 1.

### 3.3. Factors Associated with the Metabolic Syndrome (Bivariate Analysis)

The bivariate associations among sociodemographic, drug use profile, treatment modality, nutritional, and lifestyle characteristics with MS and its components are presented in Table 2 and Table 3. Considering the Bonferroni correction for multiple tests, none of the variables were significantly associated with the presence of MS (*p* ˃ 0.002 for all) (Table 2).

**Figure 2:**
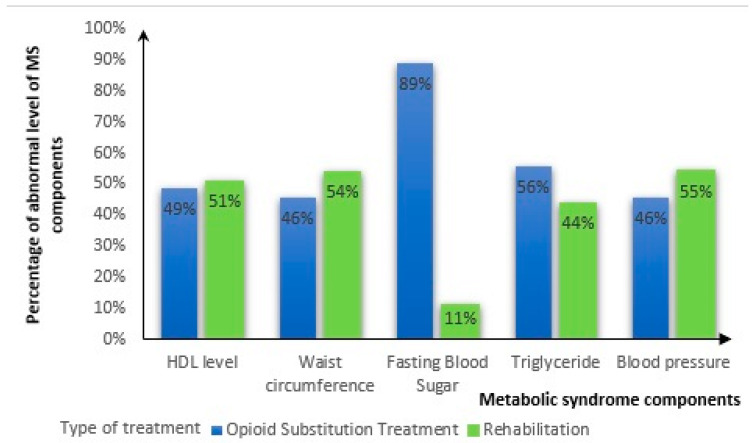
Proportions of the abnormality level of metabolic syndrome components by the type of treatment in the total sample (n = 154).; HDL-C: high-density lipoprotein cholesterol.

**Figure 3:**
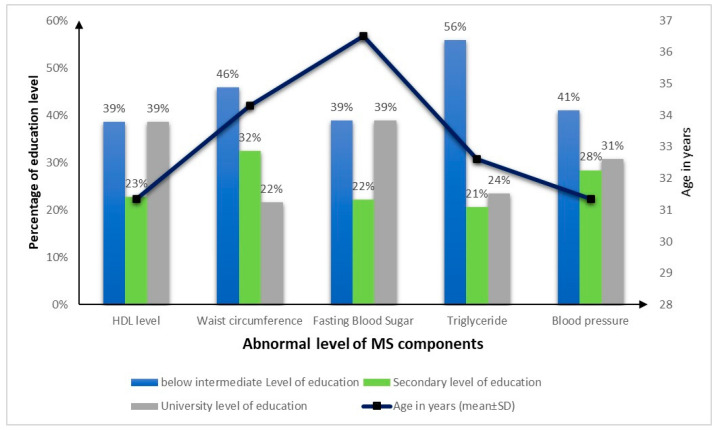
Abnormality level of metabolic syndrome components by the specific demographic variables (education level and age). FBS: fasting blood sugar; HDL: high-density lipoprotein.

Considering the component of MS and after applying the Bonferroni correction, a significantly higher proportion of participants using the OST treatment was related to the presence of FBS (Figure 2). In addition, a higher mean pre-treatment BMI was associated with normal HDL-C levels; however, a higher mean pre-treatment BMI was associated with elevated waist circumference. A significantly higher mean caloric intake per Kg of body weight was associated with normal waist circumference. Also, a higher proportion of individuals having poor sleep quality was significantly associated with elevated triglyceride levels. No significant difference was found between the component of MS and demographic factors such as education level and age (Figure 3).

### 3.4. Determinants of the Metabolic Syndrome (Multivariate Analysis)

The determinants of MS and its components are detailed in Table 3. Age was significantly associated with higher odds of being diagnosed with MS (OR = 1.070; 95% CI: 1.006–1.138), whereas the duration of current treatment and the interaction with age were not significantly associated with the presence of MS.

When considering the components of the MS as the dependent variables, pre-treatment BMI was negatively associated with abnormal HDL-C (OR = 0.886; 95% CI: 0.815–0.964). Age (OR = 1.075; 95% CI: 1.024–1.128), rehabilitation treatment (OR = 3.436; 95% CI: 1.482–7.969), the use of antipsychotics mediation (OR = 3.121; 95% CI: 1.366–7.133), a high pre-treatment BMI (OR = 1.392; 95% CI: 1.225–1.582), and weight gain (OR = 10.794; 95% CI: 3.127–37.264) were significantly associated with an elevated WC. Taking the FBS as the dependent variable, rehabilitation treatment was negatively associated with abnormal FBS (OR = 0.149; 95% CI: 0.027–0.830). Moreover, the use of antipsychotic mediation was significantly associated with elevated TG levels (OR = 2.384; 95% CI: 1.049–5.417). Finally, rehabilitation treatment was significantly associated with BP (OR = 2.984; 95% CI: 1.319–6.747); however, the use of antipsychotics (OR = 0.488; 95% CI: 0.240–0.992) was negatively associated with such odds.

## 4. Discussion

PWUD are prone to metabolic abnormalities, yet this issue has been rarely assessed within the international and national literature. We found that approximately 23% of our population of PWUD undergoing treatment for recovery had MS, with low HDL-C, elevated BP, and high WC as the predominant components. The presence of MS was higher among older patients and those with a shorter duration of treatment. Furthermore, the observed cluster of unhealthy behaviors was significant and warrants attention due to its potential long-term health impacts. Our results are comparable to those found in the literature; Vallecillo et al. [50] reported a prevalence of 29.5% of MS among Spanish individuals with heroin use disorders undergoing OST based on Adult Treatment Panel III (ATP III) criteria. Similar to our results, the predominant MS components were low HDL-C and high blood pressure; however, the participants were older than those in the present study. Moreover, the prevalence of MS among Indian males undergoing rehabilitation was 29.3% based on the International Diabetes Federation (IDF) criteria [13]. Finally, an Iranian cohort study reported that 27.3% of former opium users had MS according to ATP III criteria [14]. MS has not been widely studied among Arab populations. Regarding the Middle East and Gulf Region, there is a paucity of studies conducted on the MS among PWUD in treatment. The majority of the studies focus on the prevalence of MS in healthy individuals, with a pooled estimate of 25% in countries like Saudi Arabia, Qatar, Yemen, Kuwait, and the United Arab Emirates. Moreover, the prevalence of MS among Omani adults was around 21%, with low HDL (75.4%) as the predominant component, followed by abdominal obesity (24.6%) [51,52]. Looking at Lebanon, the prevalence of MS is higher than in the neighboring countries (31.2%), with abdominal obesity and low HDL as the predominant components. This could be attributed to the high urbanization rate coupled with westernization and changes in lifestyle [53]. Yet, it remains impossible to draw firm conclusions on where our findings stand in comparison with those of these studies since they were conducted in populations with different demographic characteristics, like age, gender, level of education, marital status, and health and disability status, compared with our sample. Information on treatment duration was also not provided by these studies. Substance abuse may increase the risk of developing the metabolic syndrome by augmenting cell damage, and excitotoxicity, reducing energy production, and lowering the antioxidant potential of the cells [15]. Furthermore, it is associated with hormonal and metabolic disorders, in which adipose tissue-derived hormones, adiponectin and leptin, play an important role in energy homeostasis and insulin resistance-associated metabolic syndrome [54].

The present study shows a positive association between the participants’ age and MS. The tendency for a higher occurrence of MS with advanced age was comparable to the results identified in other populations [55,56,57]. Data from the NHANES 2003–2006 cohort among adults 20 years of age and above in the United States show a higher prevalence of MS with age, reaching, in males, approximately 20% in those under 40 years of age, 41% in those between 40 and 59 years, and 52% in those aged 60 years and over [58]. The influence of age on the development of insulin resistance and the rising prevalence of MetS may be explained, at least in part, by the accumulation of risk factors over time, hormonal changes (particularly in women), and variations in the secretory function of the pancreatic β cells, which decreases with time [59]. Furthermore, the risk of CVD, which is associated with the presence of MS, is enhanced by accelerated arterial aging that results in endothelial dysfunction, elevated blood pressure, decreased nitric oxide bioavailability, and increased oxidative stress [60]. In contrast, the duration of treatment was negatively associated with the MS—potentially because the duration of the treatment was negatively associated with weight gain in this population [34]. A possible explanation of the decrease in weight gain at later stages of treatment lies in the consciousness of the increase in weight faced by the patients in the early stages of rehabilitation and of the difficulty in losing it at later phases. Consequently, subjects become mindful of their dietary intake and eating practices in the later stages of treatment for recovery [61]. PWUD in recovery usually regain the weight lost in active substance abuse dominantly in the early stages of treatment and even exceed the weight lost during active drug use [62]. The increase in weight and food intake in the early treatment phase may be due to the need to maintain a suitable biological weight or to the perception and usage of food as compensation for the drug due to the compromised neurobiological system [63]. Mid and later treatment phases witness improvements in disordered dietary behaviors and cravings, in which patients become concerned with the gained weight causing anxiety and preoccupation [64]. This informs future interventions to focus on newcomers and to consider the early stages of recovery, which showed to be the most critical. An increase in the BMI is accompanied by a significant increase in the occurrence of abnormal values of risk factors of metabolic syndrome, i.e., waist circumference, HDL cholesterol, and triglycerides [65]. Many of the complications of obesity arise from lipotoxicity. Adipocytes store excess free fatty acids (FFA) in the cytosol and release adipokines that are inflammatory and associated with the development of MetS and atherosclerosis. Non-adipose cells have a limited capacity to store FFA and, with exposure to excess FFA, undergo metabolic derangements, cellular dysfunction, or cell death. Weight loss that preferentially reduces metabolically active visceral adipose tissue may have a salutary effect on the components of the MS [66].

Furthermore, studies addressing the components of MS in PWUD undergoing treatment are lacking. Comparing our results with those conducted among other populations, BMI was inversely proportional to HDL-C in an obese population with a broad-spectrum BMI. This could be explained by the effect of obesity on insulin resistance and lowering HDL-C [67]. Moreover, relating BMI to the prevalence of the major risk factors for CHD in a population of white men and women showed that reduced HDL-C and hypertension were more strongly associated with higher BMI [68]. Treatment modality and, specifically, substance withdrawal in rehabilitation centers is associated with increases in BP. Opiate withdrawal can cause the autonomic nervous system to become hyperactive, leading to increased heart rate and blood pressure [69]. Moreover, this increase in BP can be attributed to the increase in vascular stiffness and cardiovascular age in opiate withdrawal. Furthermore, medications used in SUD treatments like antidepressants and anti-anxiety drugs can cause an increase in blood pressure as a side effect [70,71]. Additionally, the process of detoxification and lifestyle change is often stressful, leading to the activation of the sympathetic nervous system, which, in turn, raises blood pressure [72].

WC increases with age and is driven by gains in body weight [73]. Moreover, Walls et al. [74] showed that the increase in WC among a civilian US population was consistent across different BMI categories. These results imply that the negative health consequences related to obesity may be progressively underestimated by trends in BMI alone, whereas WC is more strongly linked to negative cardiovascular outcomes. Additionally, Mahboub et al. [34] showed a higher weight gain in rehabilitation centers compared with OST. This was attributed to the regimented meals provided at residential centers, cravings for sugary foods as a substitute for drugs, and overeating as a coping mechanism against the constraints of the strict environment that is enforced. Finally, an association can be found between a statistically significant increase in acute BP changes and the initiation of antipsychotic medications [75]. Most antipsychotic agents utilized in the treatment of schizophrenia, bipolar disorder, and other psychoses are dopaminergic antagonists that interact with the renin–angiotensin–aldosterone system, hence disrupting the regulation of BP [76]. Furthermore, Sason et al. [77] demonstrated an increase in BP among PWUD undergoing methadone treatment. This was attributed to the increase in weight observed and to the dose of methadone administered.

Looking at the nutrition and lifestyle practices, we found that dietary variables, nutrition knowledge, food addiction, physical activity level, and sleep quality were not associated with MS or any of its components. These associations remain highly understudied in the international literature, especially among PWUD. Studies on normal populations show a positive association between poor dietary behaviors, poor nutrition knowledge, and low to moderate physical activity with the prevalence of MS [78]. Furthermore, insulin resistance and individual clinical components of MS may result from sleep disturbances independently [79]. Similar to our results, obese patients looking for weight loss treatment demonstrated high food addiction frequency, which correlated with psychosocial functioning more than it did with metabolic parameters [80]. This lack of association in our sample could be explained by the fact that the variables concerning dietary intake and lifestyle practices were assessed using questionnaires depending on the participants’ recall, which may not be representative of the person’s typical intake. These variables were collected based on participants’ self-reported data. Another explanation could be that our sample size is relatively small. Thus, further studies with larger samples are needed. Furthermore, few studies highlighted the importance of healthy eating, regular exercise, and adequate sleep for PWUD to improve their physical and emotional well-being. Not addressing various risk behaviors, jeopardizes the opportunity to reduce incidences of chronic diseases in that population [62].

PWUD in treatment centers go through a major transition from a disordered lifestyle to a more disciplined one, in which they start to independently make their life choices. This transition fosters the development of healthy habits and positive behavioral changes [81,82]. Moreover, background information about a client’s physical health is vital for the treatment assessment [83]. It has been shown that when starting substance use treatment, a client’s initial health status can predict the client’s health status at later stages of treatment [84]. Furthermore, poor health status might hinder treatment engagement and can be a potential factor in relapse. It is not only by succeeding in reducing or eliminating substance use that a treatment program for SUD is considered effective but also through holistically improving clients’ lives in areas such as personal health and public health risks [85,86].

However, current research lacks comprehensive interventions targeting nutrition and health behaviors within SUD treatment, with existing studies lacking a unified conceptual framework addressing all health risk behaviors [87]. The dearth of studies in the literature tackling nutrition interventions among this population group mainly focuses on implementing nutrition and food classes to enhance healthy food choices among participants in addiction treatment facilities. This resulted in significantly greater intakes of fruits and vegetables, lower intakes of calories from sweets and desserts, and a reduction in waist circumference following the intervention, compared to the control period. Yet these results need to be confirmed in larger randomized trials [88,89]. These findings support nutrition education as an essential component of substance abuse treatment programs that can enhance treatment outcomes. Dietitians play a vital role as part of the treatment health care team promoting and encouraging the inclusion of nutrition education into substance abuse treatment programs. Furthermore, including a structured supervised routine of high frequency and long-term exercise habits or sports lifestyles for this population group in treatment centers has shown improved mood, reduced craving, improved sleep, and enhanced physical fitness [90]. Interestingly, studies show high levels of interest in incorporating exercise into treatment centers, yet low rates of involvement in exercise programs. Given this, it is important to create an individualized exercise program tailored to the patient’s preferences and tolerance for proper adherence [61,91].

Given this, implementing lifestyle interventions and health promotion programs during this pivotal phase could play a crucial role in fostering a healthier population [92]. It is imperative to focus on initiatives that aim to prevent weight gain, promote physical activity, and improve sleep quality within SUD treatment to mitigate future health complications and reduce the risk of relapse.

Based on substantial studies within this demographic group, there is a need to develop appropriate intervention strategies and public health programs to advance the nutritional status and lifestyle habits in treatment centers. Noncommunicable diseases may pose serious public health and economic burdens; however, this can be limited by addressing the challenges to a healthy lifestyle that PWUD in treatment centers face.

### 4.1. Strengths and Limitations

The present study pioneers in investigating the prevalence of MS in PWUD undergoing treatment for recovery in Lebanon. Moreover, an exhaustive sample was used, in which all participants in treatment centers that gave us permission to enter were approached. Additionally, data collection was conducted by licensed dietitians, nurses, and phlebotomists using calibrated instruments and validated tools that were appropriately translated into Arabic. Finally, biochemical parameters were analyzed in a laboratory certified by the MOPH in Lebanon.

Our study comes with some limitations. First, no conclusions regarding causality could be deduced due to the cross-sectional nature of the study. Second, female participants were not represented because few rehabilitation centers in Lebanon host females, along with the shame accompanying females upon receiving OST. Third, participants’ pre-treatment weight, BMI, sleep, and physical activity were self-reported instead of being measured. Reported data in exploratory studies are important and practical but suffer from recall bias. These parameters must be explored even further in future studies using other techniques that quantify physical activity, like motion sensors or weighing patients upon admission to treatment facilities. Furthermore, dietary intake was assessed using a single 24 h dietary recall that produced some understanding of the participants’ intake but might not describe a typical intake. Future studies using objective measures of dietary intake, energy expenditure, or three-day dietary recall are warranted to overcome substantial errors in one-day self-reported food intake. Fourth, due to the cross-sectional study design, there were no baseline data for biochemical and nutritional parameters to explore the impact of the treatment and compare our results. Fifth, the participants’ responses may have been more influenced by social desirability and thus less accurate, probably to avoid conflict with their host institution, as they were still actively engaged in the rehabilitation centers during data collection. Furthermore, psychometric validation of the Arabic versions of the translated questionnaires used in the study is needed for future use. Moreover, the lack of national data regarding the variables studied in the general Lebanese population prevented comparison against an aged-matched control group. Additionally, the social and economic hardships that Lebanon is facing impose additional challenges on the participants, especially in the OST group, whose nutrition and lifestyle factors are affected. Finally, the small sample size might not reflect the diversity of the population, and the low response rate might lead to a nonresponse bias. Hence, the extent to which our results may be generalized is limited, as it might not reflect the true population characteristics. Future studies in other countries are needed to determine whether our findings characterize PWUD treated in Lebanon exclusively or can indicate common features with other populations.

### 4.2. Practical Implications

We report a cluster of clinical factors and unhealthy behaviors that would be conducive to a future MS. Increasing awareness in PWUD undergoing treatment for recovery of the associated risks of chronic diseases and implementing intervention programs aimed at improving dietary and lifestyle practices of this population group, specifically in older participants and newcomers, are warranted. Such programs should encompass several aspects, such as weight gain monitoring, as well as food addiction treatment, while increasing nutrition knowledge and enhancing physical activity, in addition to optimizing sleep. Health promotion programs addressing unhealthy behaviors are lacking in SUD treatment facilities. PWUD in treatment centers are transitioning from a chaotic lifestyle to a disciplined one while gaining more autonomy over their life choices. Thus, they have the opportunity for good behavior modification while promoting productive habits [81,82]. During these critical times, endorsing healthy habits through lifestyle interventions and health promotion programs is vital to building a healthier population [93]. Programs should focus on monitoring weight gain, remediating food addiction, increasing nutrition knowledge, enhancing physical activity, and improving sleep. Health promotion programs tackling unhealthy behaviors, which have been proposed in this study, are lacking and are not a priority in treatment centers in Lebanon [94]. The implementation of a health promotion program and providing primary health care initiatives and screening to PWUD in treatment have to be supported by national policies with a governmental key role. Moreover, differences in treatment modalities should be accounted for in intervention programs. Treatment rehabilitation centers are ideal institutions for such intervention programs since they provide the first steps toward lifestyle changes in all aspects under controlled professional support [85,95]. As for OST centers, the implementation of such programs faces more challenges due to external factors in the patients’ lives that are beyond the control of the health care providers. The introduction of such programs has been successful, particularly those that empower individuals through education and by providing support for health improvement [96,97]. Finally, developing and implementing a health promotion intervention for treatment centers requires baseline data about dietary and lifestyle practices to identify the risks and address them. Little data on this vulnerable group are available worldwide and in Lebanon. More research and funding should be invested in this population group as they exhibited unhealthy lifestyle behaviors posing them at high risk of future health problems adding to the burden of the health care system.

### 4.3. Future Studies

Generating policies to improve the treatment of PWUD while undertaking all facets of behavioral changes requires further widespread research conducted in this population group. Thus, longitudinal studies can assist in investigating variations in lifestyle practices, biochemical indicators, and weight throughout all stages of recovery, as well as exploring their implications on the outcomes of the treatment, in addition to disease development. Furthermore, to increase the generalizability of the results, future studies must include representative samples in terms of size and gender distribution. Moreover, similar studies in other regions must be conducted to determine if this study’s results are common among PWUD undergoing recovery treatment or restricted to specific cultures. Furthermore, when designing future longitudinal studies, it is important to include measured, not reported, parameters, like dietary intake, weight and weight change, sleep, and physical activity, to better assess weight gain patterns, as well as to explore individualized needs, such as the type and duration of adopted physical activity. Additionally, while planning health promotion interventions, a crucial first step is to administer a health needs assessment. Finally, our research findings emphasize the important factors to be addressed in terms of weight gain, food addiction, nutrition knowledge, and quality sleep among PWUD in treatment centers in Lebanon.

## 5. Conclusions

This study is among the few to investigate MS among PWUD undergoing treatment for recovery. The results indicate that 23% of this population group has metabolic syndrome, putting them at risk for chronic diseases. The higher prevalence of metabolic syndrome with age and in newcomers emphasizes the need for intervention at the early stages of treatment and in older treatment seekers. The implementation of primary prevention methods, like routine metabolic screening in SUD treatment protocols, the multidisciplinary management of medical conditions by primary care providers, tailoring individualized health care plans based on each patient’s medical profile and risk factors, stress management and behavioral interventions to reduce effects on metabolic outcomes, and emphasis on continuing medical follow-ups after SUD treatments, could be recommended in drug treatment centers to improve patient outcomes. Further research on a larger sample size assessing the evolution of MS with treatment time and associated factors, including sociodemographic, nutritional, lifestyle, and treatment-related parameters across different treatment modalities, are warranted.

## Figures and Tables

**Table 1:** Demographics, medical history, and drug use history of the participants (n = 155).

	Total		OST		Rehabilitation		*p*-Value
	Mean	SD	Mean	SD	Mean	SD	
Age	32.53	8.39	33.60	8.03	30.29	8.32	**0.013**
	N	%	N	%	N	%	
Educational level							
Illiterate	9	5.8	7	8.8	2	2.6	0.320
Elementary/intermediate	55	35.5	25	31.2	30	40.0	
Secondary	45	29.0	25	31.2	20	26.7	
University	46	29.7	23	28.8	23	30.7	
Occupation							
Unemployed/Retired	72	46.5	35	43.7	37	49.3	0.125
Employed	35	22.6	21	26.3	14	18.8	
Self-employed	43	27.7	24	30.0	19	25.3	
Student	4	2.6	0	0	4	5.3	
Other	1	0.6	0	0	1	1.3	
Marital status							
Single	112	72.3	53	66.3	59	78.6	0.185
Married	32	20.6	21	26.3	11	14.7	
Divorced/separated	11	7.1	6	7.4	5	6.7	
Medications Used							
Antidepressants	35	22.6	14	17.5	21	28.0	0.118
Antipsychotic	58	37.4	28	35.0	30	40.0	0.520
Epilepsy-bipolar	33	21.3	10	12.5	23	30.7	**0.006**
Type of drug used							
Drug use only	78	50.4	29	36.3	49	65.4	**<0.001**
Drug injection only	1	0.6	0	0	1	1.3	
Drug use and injection	75	48.4	51	63.7	24	32.0	
No response	1	0.6	0	0	1	1.3	
Frequency of drug use or injection							
Up to 3 times daily	121	78.1	69	86.3	52	69.3	0.062
Once or more daily	26	16.8	9	11.3	17	22.7	
Once or more weekly	5	3.2	2	2.4	3	4.0	
Does not know or remember	1	0.6	0	0	1	1.3	
No response	2	1.3	0	0	2	2.7	
Previous treatment							
None	70	45.2	31	38.7	39	52.0	**0.005**
OST	9	5.8	5	6.3	4	5.3	
Rehabilitation	46	29.7	20	25.0	26	34.7	
Rehabilitation and OST	9	5.8	6	7.5	3	4.0	
Hospital detoxification	16	10.3	14	17.5	2	2.7	
Hospital detoxification and rehabilitation	4	2.6	4	5.0	0	0	
No response	1	0.6	0	0	1	1.3	
	**Mean**	**SD**	**Mean**	**SD**	**Mean**	**SD**	
Duration of drug use (years)	11.56	7.41	12.02	7.44	11.04	7.40	0.360
Duration of drug injection (years) (among those who reported drug injection)	7.59	6.44	7.20	6.65	8.6	5.89	0.321
Age at first drug use and/or injection (years)	16.57	3.95	17.85	5.13	16.11	4.45	**0.028**
Duration of current treatment (months)	17.72	21.38	29.07	24.33	5.62	5.65	**<0.001**
Number of previous treatment attempts	3.17	4.53	3.66	5.06	2.10	2.47	0.187

SD: Standard deviation; OST: opioid substitution therapy. Bold: significant *p* values.

**Table 2:** Bivariate analysis of sociodemographic, drug use profile, treatment modality, nutritional, and lifestyle characteristics and metabolic syndrome (n = 154).

	No Metabolic Syndrome (n = 119)	Metabolic Syndrome (n = 35)	*p*-Value
Age in years (mean ± SD)	31.15 ± 7.59	35.02 ± 10.01	0.058
Educational level			
Illiterate (%)	77.8	22.2	0.733
Elementary/intermediate (%)	72.7	27.3	
Secondary (%)	82.2	17.8	
University (%)	77.8	22.2	
Duration of drug use in years (mean ± SD)	11.38 ± 6.43	12.30 ± 10.09	0.673
Duration of drug injection in years [among those who inject drugs] (mean ± SD)	7.12 ± 6.69	9.27 ± 5.40	0.095
Type of drug use			
Drug use only (%)	75.6	24.4	0.824
Drug injection only (%)	100	0	
Drug use and injection (%)	78.4	21.6	
No response (%)	100	0	
Number of previous treatment attempts	3.30 ± 4.90	2.60 ± 2.20	0.344
Duration of current treatment in months (mean ± SD)	18.79 ± 21.86	13.23 ± 18.97	0.236
Type of treatment			
OST (%)	83.5	16.5	0.057
Rehabilitation (%)	70.7	29.3	
Current use of antidepressants, Yes (%)	73.5	26.5	0.555
Current use of antipsychotics, Yes (%)	74.1	25.9	0.471
Current use of epilepsy/bipolar medications, Yes (%)	65.6	34.4	0.077
Pre-treatment BMI in Kg/m^2^ (mean ± SD)	24.60 ± 4.18	26.89 ± 6.56	0.015
Weight change			
Weight loss (%)	90	10	0.179
No change (%)	75	25	
Weight gain (%)	74	26	
Caloric intake per Kg of body weight in Kcal/Kg (mean ± SD)	33.85 ± 19.18	28.66 ± 10.59	0.044
Protein intake per Kg of body weight in g/Kg (mean ± SD)	1.05 ± 0.64	1.01 ± 0.61	0.590
Percentage of carbs from total calories (mean ± SD)	49.41 ± 10.86	48.82 ± 8.90	0.772
Percentage of added sugar from total calories (mean ± SD)	2.92 ± 3.66	1.98 ± 2.94	0.091
Percentage of fat from total calories (mean ± SD)	38.66 ± 10.59	37.47 ± 7.90	0.544
Fiber intake in g (mean ± SD)	22.37 ± 12.52	21.86 ± 9.53	0.670
Food addiction			
No diagnosis (%)	83.1	16.9	0.061
Diagnosis met (%)	70.3	29.7	
Nutrition knowledge			
Poor knowledge (%)	79.4	20.6	0.375
Good knowledge (%)	73.1	26.9	
Sleep quality index			
Good sleep quality (%)	81.1	18.9	0.510
Poor sleep quality (%)	75.9	24.1	
Physical activity level			
Low (%)	79.7	20.3	0.783
Moderate (%)	75	25	
High (%)	75	25	

SD: Standard deviation; OST: opioid substitution therapy; BMI: body mass index.

**Table 3:** Logistic regression of the determinants of the metabolic syndrome and its components.

	*p*-Value	95% CI		
		OR	Lower	Upper
Model 1: Metabolic Syndrome				
Age (years)	**0.031**	**1.070**	**1.006**	**1.138**
Duration of current treatment (months)	0.621	0.971	0.865	1.091
Interaction between age and duration of treatment	0.984	1.000	0.997	1.003
Caloric intake per Kg of body weight	0.292	0.984	0.956	1.014
Variables included in the model: age (years), duration of current treatment (months); caloric intake (Kcal/Kg).				
Model 2: High-Density Lipoprotein cholesterol				
Educational level (elementary vs. illiterate *)	0.488	1.732	0.367	8.164
Educational level (secondary vs. illiterate *)	0.742	1.301	0.272	6.221
Educational level (university vs. illiterate *)	0.074	4.260	0.868	20.904
Pre-treatment BMI in Kg/m^2^	**0.005**	**0.886**	**0.815**	**0.964**
Physical activity level (moderate vs. low *)	0.642	0.818	0.352	1.904
Physical activity level (high vs. low *)	0.155	1.901	0.784	4.610
Sleep quality index (poor vs. good *)	0.567	1.268	0.562	2.865
Food addiction (diagnosis met vs. not *)	0.428	1.091	0.879	1.354
Nutrition knowledge (good vs. poor *)	0.720	1.147	0.541	2.430
Variables included in the model: educational level; pre-treatment BMI (Kg/m^2^); physical activity (reference: low); sleep quality index (reference: good); food addiction (reference: no addiction); nutrition knowledge (reference: poor).				
Model 3: Waist Circumference				
Age	**0.003**	**1.075**	**1.024**	**1.128**
Type of treatment use (rehabilitation vs. OST *)	**0.004**	**3.436**	**1.482**	**7.969**
Current use of antipsychotics (yes vs. no *)	**0.007**	**3.121**	**1.366**	**7.133**
Pre-treatment BMI in Kg/m^2^	**<0.001**	**1.392**	**1.225**	**1.582**
Weight change (no change vs. weight loss *)	0.177	2.660	0.644	10.986
Weight change (weight gain vs. weight loss *)	**<0.001**	**10.794**	**3.127**	**37.264**
Food addiction (diagnosis met vs. not *)	0.523	0.923	0.723	1.179
Variables included in the model: age (years); type of treatment (reference: OST); current use of antipsychotics (reference: no use); pre-treatment BMI (Kg/m^2^); weight change (reference: weight loss); food addiction (reference: no addiction).				
Model 4: Fasting Blood Sugar				
Age	0.082	1.055	0.993	1.121
Type of treatment use (rehabilitation vs. OST *)	**0.030**	**0.149**	**0.027**	**0.830**
Physical activity level (moderate vs. low *)	0.271	0.406	0.081	2.023
Physical activity level (high vs. low *)	0.829	0.820	0.135	4.979
Variables included in the model: age (years); type of treatment (reference: OST); physical activity (reference: low).				
Model 5: Triglycerides				
Food addiction (diagnosis met vs. not *)	0.429	0.899	0.691	1.170
Sleep quality index (poor vs. good *)	0.053	3.520	0.982	12.621
Current use of antipsychotics (yes vs. no *)	**0.038**	**2.384**	**1.049**	**5.417**
Pre-treatment BMI in Kg/m^2^	0.097	1.071	0.988	1.160
Variables included in the model: food addiction (reference: no addiction); sleep quality index (reference: good); current use of antipsychotics (reference: no use); pre-treatment BMI (Kg/m^2^).				
Model 6: Blood Pressure				
Food addiction (diagnosis met vs. not *)	0.896	0.987	0.810	1.202
Physical activity level (moderate vs. low *)	0.298	1.555	0.678	3.566
Physical activity level (high vs. low *)	0.755	0.861	0.337	2.204
Nutrition knowledge (good vs. poor *)	0.708	1.150	0.555	2.383
Sleep quality index (poor vs. good *)	0.223	0.615	0.282	1.344
Type of treatment use (rehabilitation vs. OST *)	**0.009**	**2.984**	**1.319**	**6.747**
Current use of antipsychotics	**0.048**	**0.488**	**0.240**	**0.992**
Pre-treatment BMI in Kg/m^2^	0.139	1.060	0.981	1.144
Caloric intake per Kg of body weight	0.387	0.991	0.972	1.011

*: Reference group. Variables included in the model: food addiction (reference: no addiction); physical activity (reference: low); nutrition knowledge (reference: low); sleep quality index (reference: good); type of treatment (reference: OST); current use of antipsychotics (reference: no use); pre-treatment BMI (Kg/m^2^); caloric intake ((Kcal/Kg). OR: odds ratio; CI: confidence interval; HDL-C: high-density lipoprotein cholesterol; BMI: body mass index; Bold: significant *p* values.

## Data Availability

The group investigated is a vulnerable population, and all information is confidential. Thus, all data are kept at the Lebanese International University, where this study was conducted.

## Supplementary Materials

The following supporting information can be downloaded at https://www.mdpi.com/article/10.3390/clinpract14060210/s1: Table S1. Weight change and anthropometric measurements of the participants (n = 155). Table S2. Biochemical parameters of the participants (n = 155).
