# Supplementary material for: Metabolic Syndrome as a Risk Factor Among Lebanese Patients with Substance Use Disorder Undergoing Treatment for Recovery Through Rehabilitation or Opioid Substitution Treatment
Source: Clin Pract. 2024 Dec 10;14(6):2661–80. doi: 10.3390/clinpract14060210 (PMC11674342; doi:10.3390/clinpract14060210)
Supplement: Supplementary file 1 [file clinpract-14-00210-s001.zip › clinpract-3298429-supplementary.pdf]

Table S1. Biochemical parameters of the participants (n=155)

	OST		Rehabilitation		p-value	Total	
	**Mean**	**SD**	**Mean**	**SD**		**Mean**	**SD**
**FBS (mg/dl)**	94.05	16.81	85.53	7.54	**<0.001**	89.95	13.83
**HDL-C (mg/dl)**	43.06	11.85	43.81	12.89	0.708	43.42	12.33
**TG (mg/dl)**	119.46	69.67	133.81	156.89	0.459	126.37	119.59
**Total Cholesterol (mg/dl)**	188.94	53.36	188.07	38.06	0.908	188.52	46.49
**LDL-C (mg/dl)**	115.48	44.41	114.07	31.42	0.822	114.80	38.60

SD: Standard deviation; OST: Opioid substitution therapy; FBS: Fasting blood sugar; HDL-C: High-density lipoprotein-cholesterol; TG: Triglycerides; LDL-C: Low-density lipoprotein-cholesterol.

Table S2. Anthropometric measurements of the participants (n=155)

	OST		Rehabilitation		p-value	Total	
	**Mean**	**SD**	**Mean**	**SD**		**Mean**	**SD**
**Waist circumference (cm)**	91.16	13.84	94.42	12.56	0.128	92.75	13.29
**Systolic blood pressure (mmHg)**	123.44	12.21	127.85	15.31	**0.048**	125.6	13.93
**Diastolic blood pressure (mmHg)**	75.00	11.47	78.99	15.94	0.075	76.93	13.91
**BMI (Kg/m^2)**	26.97	5.37	27.57	4.62	0.453	27.41	4.99

SD: Standard deviation; OST: Opioid substitution therapy; BMI: Body mass index.
